# Supplementary material for: Structure and Function of the Zinc Binding Protein ZrgA from Vibrio cholerae
Source: Int J Mol Sci. 2022 Dec 29;24(1):548. doi: 10.3390/ijms24010548 (PMC9820375; doi:10.3390/ijms24010548)
Supplement: Supplementary file 1 [file ijms-24-00548-s001.zip › ijms-2080559-supplementary.pdf]

Supplementary Information

Supplementary Table S1. Strains used in this study.

Strain/plasmid	Genotype/ description	Source
Strains		
Vc O395	*V. cholerae* Wild-type, Sm^r	A gift from Dr. Fitnat Yildiz at the University of California Santa Cruz
	Vc 0395 mutant, Δ*znuA*, Sm^r	This work
	Vc O395 mutant, Δ*znuA zrgA* Δ124-184, Sm^r	This work
	Vc O395 mutant, Δ*znuA*, Δ*zrgA* Sm^r	This work
Sm10 λ pir	*E. coli,* with derivative RP4 plasmid integrated, containing the λ pir for replication of R6K suicide vector, for integration into *V. cholerae.*	A gift from Dr. Fitnat Yildiz at the University of California Santa Cruz
Plasmids		
pCDF-Duet1	Expression vector, Sm^r	Novagen
	pCDF-Duet1 with *V. cholerae zrgA*, Sm^r	This work
	pCDF-Duet1, with *V. cholerae zrgA* mutation lacking *Δ124-184*, Sm^r	This work
	pCDF-Duet1, with *V. cholerae zrgA* mutation lacking *Δ124-183*, Sm^r	This work
	pCDF-Duet1, with *V. cholerae zrgA* mutation lacking *Δ124-180*, Sm^r	This work
pGP704sacB28	pGP704sacB28, R6K suicide plasmid, Ap^r	A gift from Dr. Fitnat Yildiz at the University of California Santa Cruz

	pGP704sacB28, with upstream and downstream flanking regions comprising 3 amino acids forward and reverse of *znu*A gene along with 500-600bp flanking gene, Amp^r	This work
	pGP704sacB28, with upstream and downstream 600bp flanking sequences of *zrgA*, Amp^r	This work
	pGP704sacB28, with upstream and downstream 600bp flanking sequences of *zrgA* flexible loop region *124-184*, Amp^r	This work

Supplementary Table S2. Primers used in this study.

Primer	Primer sequence 5′ to 3′
VcholZrgA_pdcf_fow	gtataagaaggagatatacatatgcattacccaacaatgattg
VcholZrgA_pcdf_rev	gtttctttaccagactcgagggtttacagtttgataagcgtttg
ZrgA_Del 123-184 FWD	ggctcattcaccgctcaatatcagttccac
ZrgA_Del 123-184 REV	accataagcgtggctgtgttggtactcttc
ZrgA_del_123-180 FWD	gagcatcaacacggctcattcaccgctcaa
ZrgA_del_123-183 FWD	cacggctcattcaccgctcaatatcagttc
Vc_ZnuA_Del_A	gatccacgaagcttcccatggaagcaagtcgggtctttgtaataaagc
Vc_ZnuA_Del_B2	taagattgggacaaacacgctctcgataacatggtcgccaaact
Vc_ZnuA_Del_C2	tggcgaccatggttatcgagagcgtgtttgtcccaatcttaagtcg
Vc_ZnuA_Del_D2	tagaaccggtgacgtcaccacaactcgtgttgctcaaaagcc
Vc_ZrgA_del_A	atccacgaagcttcccatggttccaacagtgattgcgctttgt
Vc_ZrgA_del_B	tgttttattaggagtttttctgccaccgctcggga
Vc_ZrgA_del_C	acctatcccgagcggtggcagaaaaactcctaataaaacaaaaaa
Vc_ZrgA_del_D	tatctagaaccggtgacgtcacccatcaatcacatcgggtga
ZrgA_Mut_124-184_A	gatccacgaagcttcccatggcaagcggcttaagtcactgc
ZrgA_Mut_124-184_B2	aacacagccacgcttatggtggctcattcaccgctcaatat
ZrgA_Mut_124-184_C2	tattgagcggtgaatgagccaccataagcgtggctgtg
ZrgA_Mut_124-184_D2	tagaaccggtgacgtcaccaccaccacaagcaagggttga
FWD_Vc_ZnuA_Seq	tgttgataaaaagccagcgtgtctcggc
REV_Vc_ZnuA_Seq	cattagtcgctaaagcccatttcgagact
Vc_ZrgA_Seq_FWD	tcacgggtttattcagtaaagcgcttggc

Vc_ZrgA_Seq_REV	cttgattcagcaaaatctgtaatcgcccc
FWD_Vc_ZrgA_mut_Seq	gttggaaaatatagcctatatggtcggcg
REV_Vc_ZrgA_mut_Seq	ggcctaaaaaccagtcagtggatattgcc

Table S3. Properties of refined zinc sites and ligands.

Site	Chain	Occ.	B-factor	Anom. Density (rmsd, σ)	Ligand* (Distance, Å)
1	A	1.0	32.9	12.1	H35 Nε (2.0)
					H36 Nε (2.0)
					E66 Oε1 (2.1)
					E66 Oε2 (2.1)
					H_2O (2.1)
	B	1.0	40.0	10.5	H35 Nε (2.1)
					H36 Nε (2.0)
					E66 Oε1 (2.1)
					E66 Oε2 (2.1)
					H_2O (2.1)
	C	1.0	31.3	11.5	H35 Nε (1.9)
					H36 Nε (2.0)
					E66 Oε1 (2.0)
					H_2O (2.1)
	D	1.0	43.5	12.1	H35 Nε (1.9)
					H36 Nε (1.9)
					E66 Oε1 (2.1)
					E66 Oε2 (2.1)
	E	0.43	81.1	4.7	H35 Nε (2.3)
					H36 Nε (1.8)
					E66 Oε1 (2.0)
					E66 Oε2 (2.1)
	F	0.75	43.1	7.9	H35 Nε (1.9)
					H36 Nε (2.0)
					E66 Oε1 (2.1)
					E66 Oε2 (2.1)
					H_2O (2.1)
2	A	1.0	33.5	10.3	E83 Oε1 (2.0)
					H86 Nε (2.0)
					H87 Nε (2.0)
					E79′ (D) Oε1 (2.1)
					E79′ (D) Oε2 (2.1)
	B	1.0	39.6	9.8	E83 Oε1 (2.0)
					H86 Nε (1.9)
					H87 Nε (1.9)

					E79″ (F) Oε1 (2.2)
					E79″ (F) Oε2 (2.2)
	C	1.0	38.5	9.0	E83 Oε1 (2.0)
					H86 Nε (2.0)
					H87 Nε (1.9)
					E79′ (E) Oε1 (2.1)
					E79′ (E) Oε2 (2.1)
	D	1.0	33.4	11.1	E83 Oε1 (2.1)
					E83 Oε2 (2.1)
					H86 Nε (2.0)
					H87 Nε (2.0)
					E79′ (A) Oε1 (2.1)
					E79′ (A) Oε2 (2.1)
	E	1.0	33.2	9.3	E83 Oε1 (2.1)
					H86 Nε (1.9)
					H87 Nε (2.0)
					E79′ (C) Oε1 (2.1)
					E79′ (C) Oε2 (2.1)
	F	1.0	49.4	7.2	E83 Oε1 (2.0)
					H86 Nε (2.0)
					H87 Nε (2.0)
					E79″ (B) Oε1 (1.7)
					E79″ (B) Oε2 (2.7)
3	A	1.0	34.6	10.9	H49 Nδ (2.1)
					H194 Nε (2.1)
					E196 Oε1 (2.1)
					E196 Oε2 (2.1)
					H118′ (D) Nδ (2.0)
	B	0.72	51.4	5.5	H49 Nδ (2.0)
					H194 Nε (2.3)
					E196 Oε1 (2.1)
					H_2O (2.1)
	C	0.35	54.0	4.2	H194 Nε (2.0)
					E196 Oε1 (2.1)
	D	0.43	52.8	3.0	H194 Nε (2.0)
					E196 Oε1 (2.1)
	E	0.57	60.0	5.3	H49 Nδ (2.3)
					H194 Nε (2.0)
					E196 Oε1 (2.1)
					E196 Oε2 (2.1)
					H118′ (C) Nδ (2.1)
	F	0.89	63.0	6.6	H49 Nδ (2.0)
					H194 Nδ (2.0)
					E196 Oε1 (2.0)
3b	C	0.50	55.1	3.4	H194 Nδ (2.0)
					D50 Oδ1 (2.1)

					D50 Oδ2 (2.1)
	D	0.67	51.3	7.3	H194 Nδ (1.7)
					D50 Oε1 (2.1)
4	B	0.86	62.2	4.5	H67 Nε (2.1)

*Ligands from another chain in the asymmetric unit are indicated by (‘). Those from a symmetry related chain are indicated by (“). The chain ID is indicated in parentheses after the residue name.

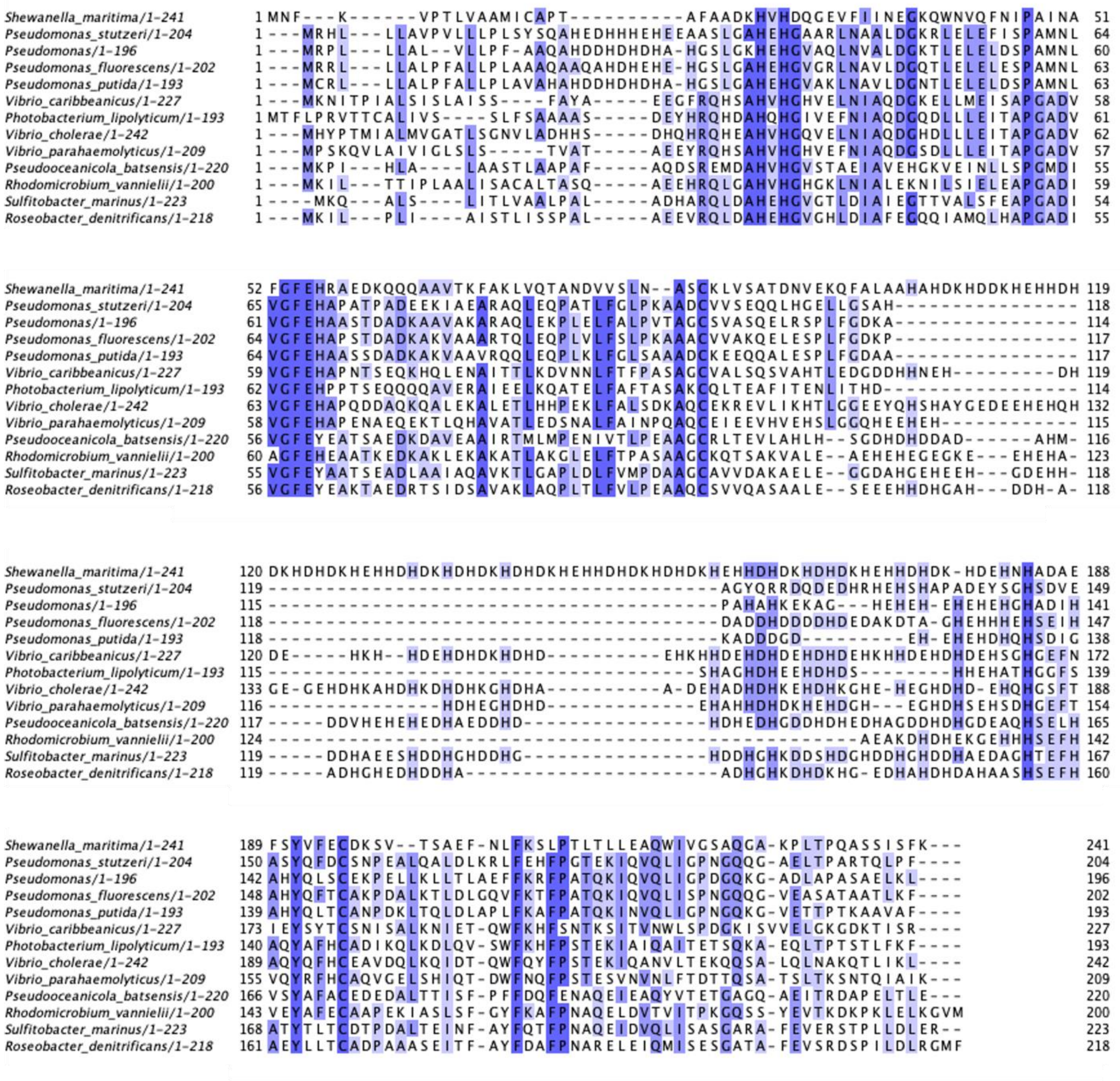

Supplementary Figure S1. Multiple sequence alignment of ZrgA homologues

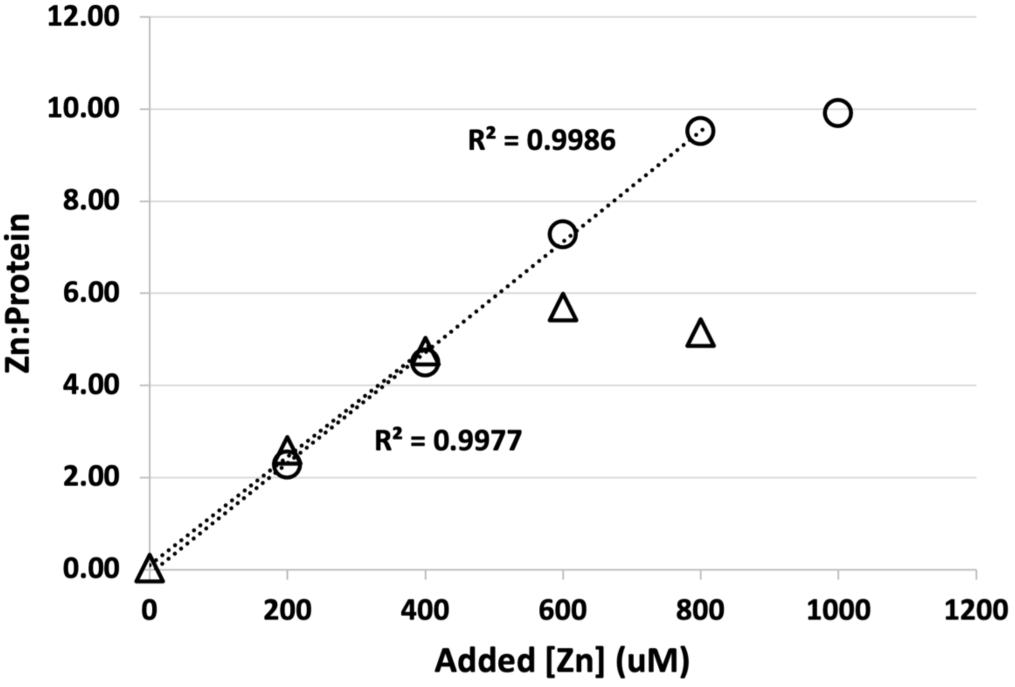

Supplementary Figure S2. Zinc binding to WT (circles) and Δ124-184 ZrgA (triangles) as determined by incubation of protein with varying concentrations of zinc followed by desalting and zinc quantitation by ICP-OES. Linear fits are applied to the first 5 data points in for WT and the first 3 for Δ124-184 ZrgA with R^2 values indicated.

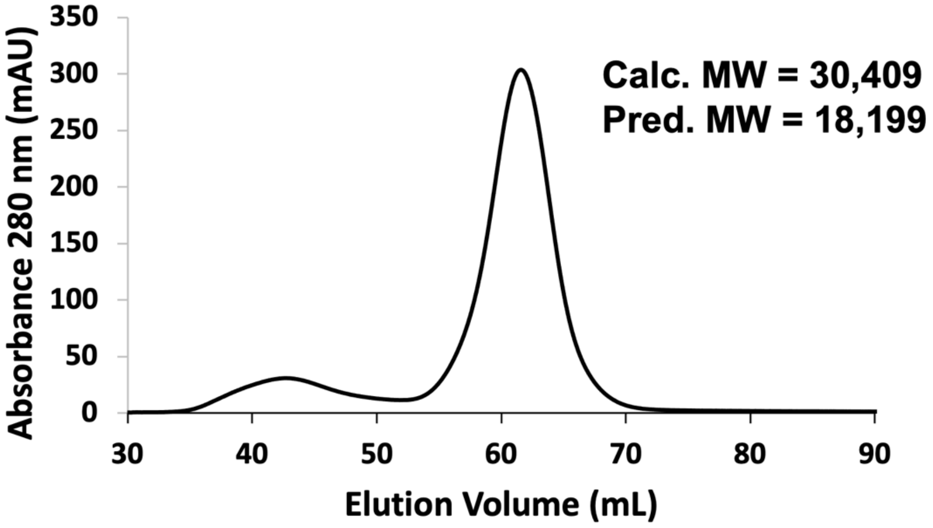

Supplementary Figure S3. Size exclusion chromatogram showing purification of ZrgA Δ124-184 and the molecular weights calculated from the elution time (*calc. MW*) and the primary sequence (*pred. MW*). The elongated structure of ZrgA likely results in a larger apparent MW than expected for a monomer that is nonetheless too small for a dimer.

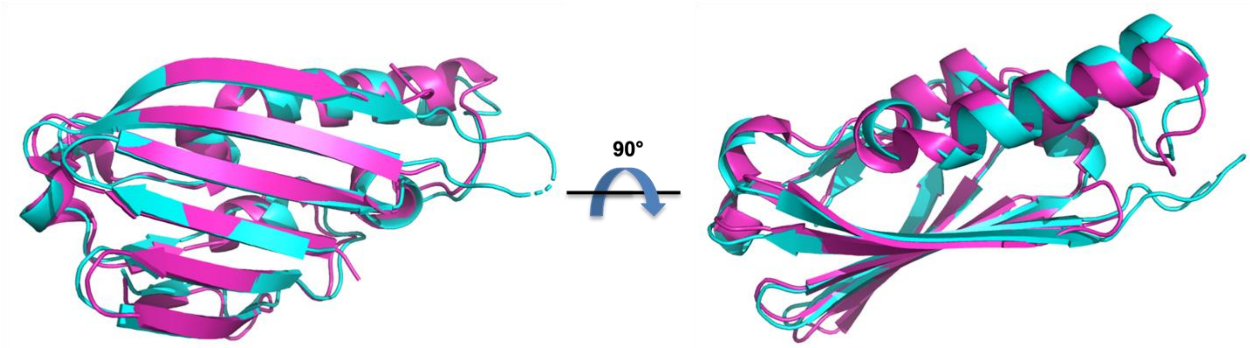

Supplementary Figure S4. Alignment of crystal structures of Apo PA4063 (PDB ID: 7AHW, magenta) and Apo Δ124-184 ZrgA (cyan). Figure created with Pymol version 2.5.4, Schrödinger, LLC.

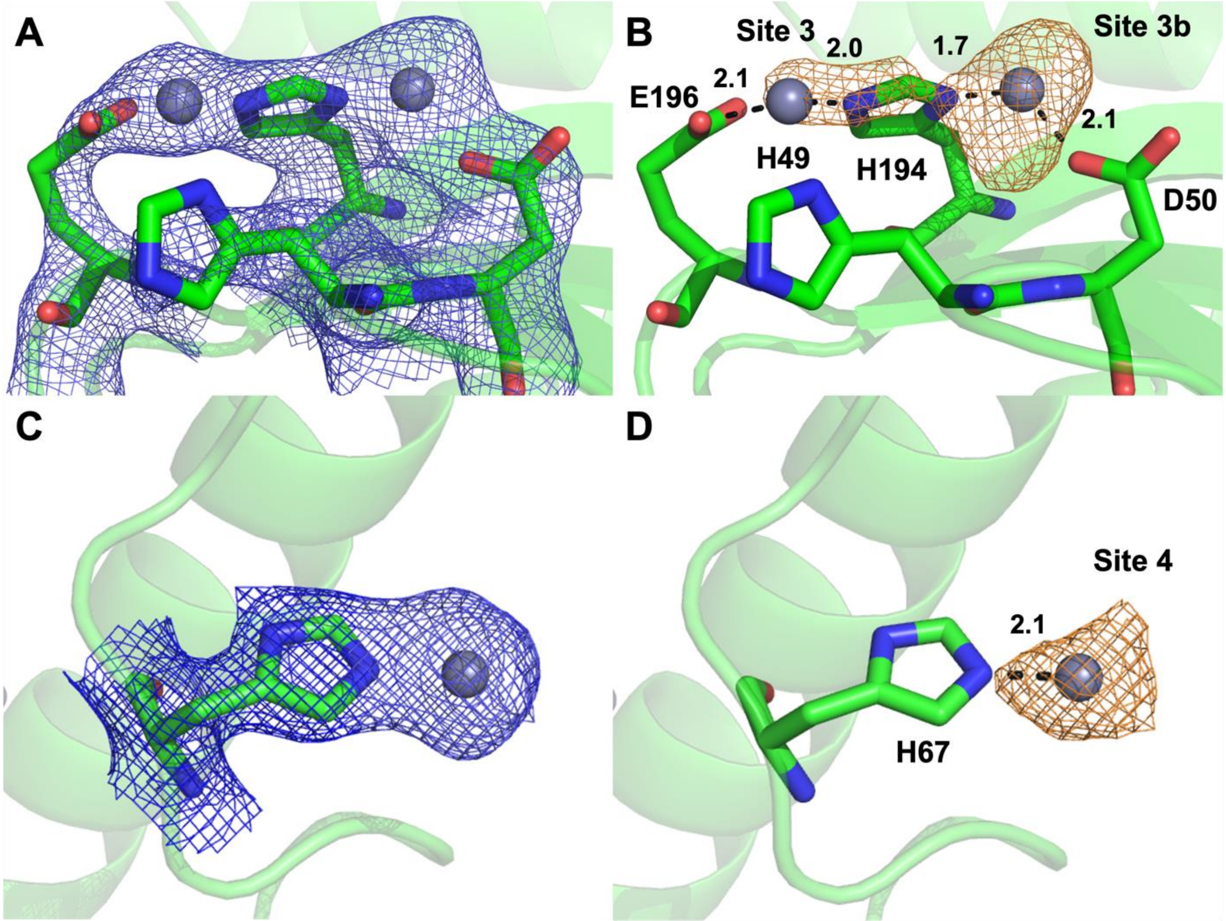

Supplementary Figure S5. Zinc binding sites 3b (A and B) and 4 (C and D) in Δ124-184 Zrg showing 2Fo-Fc density contoured at 1.0σ (blue mesh, A and C) or anomalous difference density contoured at 2.5σ and zinc ligands with bond distances indicated in Å (orange mesh, B and D). Sites 3 and 3b are shown for the D chain, and site 4 is shown on the B chain. Figure created with Pymol version 2.5.4, Schrödinger, LLC.
